# Psychometric validation of the collective asset Utu: associations with coping strategies and resilience during adolescence

**DOI:** 10.1186/s41256-023-00303-4

**Published:** 2023-05-18

**Authors:** Megan Cherewick, Ronald E. Dahl, Daphna Rubin, Jenn A. Leiferman, Prosper F. Njau

**Affiliations:** 1 Department of Community and Behavioral Health, Colorado School of Public Health, University of Colorado Anschutz, 13001 East 17th Place, Aurora, CO 80045 USA; 2 Institute of Human Development, University of California Berkeley, 2121 Berkeley Way West, Berkeley, CA 94720-1690 USA; 3 Health for a Prosperous Nation, P.O. Box 13650, Dar es Salaam, Tanzania

**Keywords:** Risk, Resilience, Adolescents, Orphans, Collective Assets, Utu

## Abstract

**Background:**

Utu is a Kiswahili term with a long history of cultural significance in Tanzania. It conveys a value system of shared, collective humanity. While variants of Utu have been studied in other contexts, a measure of Utu that captures this important collective asset has not been developed in Tanzania. The aims of this study were to (1) examine dimensional constructs that represent Utu, (2) validate a measurement scale of Utu for use with adolescents, (3) examine differences between orphan and non-orphan adolescents in self-reported Utu and, (4) examine structural paths between adverse life experiences, coping strategies, Utu, and resilience.

**Methods:**

This study collected survey data from adolescents from three districts in peri-urban Tanzania in two samples: 189 orphan adolescents ages 10–17 in May 2020 and 333 non-orphan adolescents ages 10–14 in August 2020. Confirmatory factor analysis was used to validate the hypothesized factor structure of the developed Utu measure. Structural equation models were used to examine path associations with adverse life experiences, coping and resilience.

**Results:**

The five dimensional constructs comprising the Utu measure included Resource Sharing, Group Solidarity, Respect and Dignity, Collectivity, and Compassion. Confirmatory factor analysis of the Utu measure demonstrated excellent fit (CFI = 0.98; TLI = 0.97; SRMR = 0.024; RMSEA = 0.046) and internal consistency (α = 0.94) among adolescents in this study. Positive, significant associations were found between Utu and coping (β = 0.29, p < 0.001) and Utu and intra/interpersonal and collective resilience (β = 0.13, p < 0.014). Utu was not significantly associated with adverse life experiences, age or gender.

**Conclusions:**

A five-dimensional measurement scale for Utu was validated in a sample of orphan and non-orphan adolescents in Tanzania. Utu is a collective asset associated with higher levels of reported resilience in both orphan and non-orphan adolescent populations in Tanzania. Promoting Utu may be an effective universal public health prevention approach. Implications for adolescent programming are discussed.

## Background

Emphasizing the collective wellbeing over the individual is central in much of sub-Saharan Africa. *Utu*, is an ideology, a philosophy, and a multidimensional value system. Utu encompasses respect for all humans, dignity, resource sharing, solidarity, kindness, compassion and empathy. *Utu* comes from the Kiswahili word, Mtu, meaning human being. Utu represents ideals of humanity that are closely aligned with moral concepts of goodness [1]. The word, Utu, has a Bantu base; which makes it translatable in most sub-Saharan languages. For example, variants of Utu include; ubuntu in South Africa, unhu in Zimbabwe, kimuntu in the Democratic Republic of Congo and Maaya in Burkina Faso [2, 3]. Research shows that Ubuntu, the similar term used by Xhosa people of South Africa, has been defined as, “an African value system that means humanness, which is characterized by caring, sharing compassion, communocracy and related predispositions” [4]. African proverbs further elucidate Utu as follows: “each individual’s humanity is expressed in relation with others” [5], “a person can only be a person through others” [6], and “I am because you are- I can only be a person through others” [7]. Broodryk (2002) argues that the concept of Ubuntu is an ancient African worldview based on values of caring, sharing, respect, compassion and social responsibility [8]. Desmond Tutu argues that Ubuntu, is the essence of being human [9].

In Tanzania and many sub-Saharan African countries, a person is considered to have Utu (or variants of Utu) if they express compassion, reciprocity, dignity, harmony and humanity to strengthen communities and celebrate goodness in others [10]. Other scholars emphasize the overarching commonality of empathy and associated concepts such as emotional contagion, sympathy and compassion [11, 12]. Utu provides a sense of self-assurance that stems from knowing that one belongs to a part of a greater whole, and that all people and their wellbeing are interconnected. This philosophy necessitates compassion when others are diminished and shared joy when others succeed [13].

Previous studies of Utu are limited to specific disciplines. Research on Utu in social work and education states that Utu embodies the concept of mutual understanding and the active appreciation of the value of human differences and oneness [10]. In other words, humans accept differences when they acknowledge that all humans possess Utu. The research also posits that out of the values of Utu and human dignity flow the practices of compassion, kindness, altruism and respect, which are at the very core of making schools places where the culture of teaching and the culture of learning thrive [14]. Other researchers have highlighted its importance in social work as Utu demonstrates intention for communal relationality, communal ideals, and human excellence. According to social work researchers Jacob Mugumbate and Andrew Nyanguru (2013), the related concept of Ubuntu represents the worldviews of populations of Sub-Saharan Africa, transmitted from generation to generation through observation, experience, language and art [9]. Utu is grounded in religious and philosophical traditions that have created a humanist framework that values kindness and reciprocity [15]. Scholars have argued that Utu and related concepts have been diminished in response to colonialist histories and capitalism that emphasize the individual over the collective [16]; in Tanzania elders echo the concern that younger generations are losing Utu.

Utu is a collective asset that shares some similarities to the concept of collective efficacy. Collective efficacy is defined as "a group's shared belief in its conjoint capabilities to organize and execute the courses of action required to produce given levels of attainments" [17, 18]. One study that validated a community collective efficacy scale in an African context emphasizes the "shared capacities of the group in which people participate regarding joint activities and the successful achievement of these activities as a group" [19]. However, unlike collective efficacy, achievement or the production of 'given levels of attainment' does not fully capture the concept of Utu, because Utu also captures a value system and associated behaviors that include a philosophical value system guiding interpersonal relationships to demonstrate the dignity and respect that all humans deserve [15]. Also, while studies have shown that collective efficacy is a mediator in the pathway between stress and resilience, it is unknown whether Utu plays a similar or different mediating role in between stressful life experiences and resilience [20–22].

Researchers and practitioners have increasingly focused on resilience-promoting interventions that promote protective factors and reduce risk factors for health and wellbeing [23, 24]. Diversity in approaches to interventions seeking to enhance resilience have varied because as a construct, resilience is dynamic, multifactorial, and includes each level of the social ecological model [25]. Both internal factors (e.g. character strengths, coping flexibility) and external (e.g. family, social and community environments) protective factors enable individuals to overcome adversity [25]. Resilience-focused interventions often seek to target multiple protective factors but have varied in implementation approach including differences in delivery modality (e.g. family, school or community based interventions), length, frequency, and duration. Universal, school-based interventions have been evaluated in many global contexts. A systematic review of resilience focused interventions targeting child and adolescent mental health delivered in schools found that for of the 13 adolescent trials included in meta-analysis, resilience-focused interventions were effective for internalizing problems including depressive and anxiety symptoms [25]. Globally, studies have identified protective factors for mental health resilience including social skills and social support [26, 27], positive personality traits [26, 28, 29], family attachment and cohesion [26, 28], coping flexibility [27, 30, 31], strong morality and faith [27, 28], as associated with lower levels of internalizing and externalizing symptoms. While resilience research has received greater focus in recent decades, less research has focused on community level protective factors (e.g. community cohesion, collective efficacy) associated with mental health resilience.

The current study seeks to understand if higher levels of Utu promote resilience. It is hypothesized that those with a strong sense of the collective asset, Utu, may demonstrate greater levels of resilience defined as "the capacity of individuals to navigate their way to the psychological, social, cultural and physical resources that sustain their well-being" [32]. However, it is unknown whether capacity for Utu is associated with stress or is a protective factor unrelated to stress exposure. If Utu promotes resilience independently of stress, it holds value as an independent mechanistic pathway that can be particularly useful for universal prevention programs aimed at promoting resilience. While previous has sought to identify protective factors at each level of an individual’s social ecology, comparatively less research has focused on assessing protective factors, such as Utu, at the community level. Research is needed to validate a measure designed to capture the key dimensions of Utu. Understanding the dimensional structure of Utu will enhance precision in measurement of this valuable collective asset and facilitate use of Utu in programs designed to effect change at the population level.

The aims of this study are to (1) examine dimensional constructs that represent Utu, (2) validate a measurement scale of Utu for use with adolescents, (3) examine differences between orphan and non-orphan adolescents in self-reported Utu and, (4) model structural paths between adverse life experiences, coping strategies, Utu, and resilience.

## Methods

### Setting and study design

Tanzania is a lower middle income country with approximately 61 million and 44.9% of the population living in poverty [33]. Dar es Salaam has a population growth rate of 5.6%, which is above the national population growth rate of 2.9% and is driven by migration of citizens from rural areas seeking employment [34].

### Non-orphan context

Tanzania is experiencing a surge in the adolescent population, with approximately half of the population younger than 17.5 years and 47% younger than 15 years; it is expected that these the number of adolescents in Tanzania will double by 2055 [35–37]. The Global Out of School Children Study estimated that there were approximately 3.5 million children of school age that were not enrolled in school [38]. A study of child poverty in Tanzania indicated that 74% of children were affected by multidimensional poverty with 29% of households below the poverty line [39]. While the introduction of free primary education began in 2001 and resulted in higher secondary education enrollment, the transition rates to secondary school indicate gender disparities with 21% of boys vs. 16% of girls enrolling in secondary school [40, 41]. Moreover, studies estimate that 1 in 4 adolescent girls ages 15–19 had begun childbearing, a 4% increase in teenage pregnancy since 2010 [42].

### Orphan context

Sub-Saharan Africa is home to approximately 47 million orphaned children with an estimated 3.0 million orphans in Tanzania as of 2019 [43]. Studies indicate that orphans in low-resource contexts often experience higher levels of stress and adverse life experiences in comparison to non-orphans including physical neglect, emotional neglect, emotional abuse, and physical abuse [44–46]. Sources of stress include adopting caretaker roles, instability in housing and school attendance, separation from siblings, and heightened vulnerability to child labor and exploitation [47, 48]. Research from Tanzania has found orphans had greater internalizing problems compared to non-orphans (p < 0.001) and approximately 35% had contemplated suicide [49]. These results are similar to findings comparing orphans to non-orphans in Uganda that indicated that orphans had higher levels of depression and lower levels of hope in comparison to non-orphans [50].

### Survey measures

#### Utu scale measure development

The development of the Utu scale measure was completed in close collaboration with local research partners including Health for a Prosperous Nation, a Tanzanian NGO that administered surveys to adolescents and Ubongo Kids, a Tanzania-based organization that develops engaging and locally relevant digital content for children in Africa. Ubongo Kids’ edutainment content is disseminated via TV and radio episodes to over 6.4 million East African households weekly and aired on national television via the Tanzanian Broadcasting Corporation (TBC) every Saturday. Ubongo Kids’ *edutainment* (a combination of educational and entertainment content) video content target areas of adaptive social emotional mindsets and skills designed to be culturally relevant to children and adolescents in Tanzania.

First, to generate a culturally relevant pool of items for the Utu scale, the team conducted formative, qualitative research that explored how youth, adults and community members defined the concept of Utu. Ubongo Kids in partnership with researchers at University of California Berkeley collected data in May 2019. Qualitative research included 42 in-depth interviews and 4 focus groups, resulting in a total qualitative sample of 92 participants. Two focus groups were conducted with children and adolescents, one with female adults and one with male adults. Adult participants included church leaders, teachers, parents, and older siblings.

The formative research explored essential dimensions of Utu described by participants. These descriptions were then compared to those found in in-depth literature reviews. Researchers found the results of the formative research aligned with the literature reviews in describing concepts of Utu. Results indicated five core dimensions comprising the construct of Utu in Tanzania. These constructs included Resource Sharing, Respect and Dignity, Solidarity, Collectivism and Compassion. To measure these constructs, researchers adapted items from previously validated scales of Ubuntu used in South Africa [51, 52]. They also added the compassion dimension using select items from the validated compassionate engagement scale [53, 54]. Items were slightly adapted to ensure cultural relevancy and appropriateness for adolescent populations. Response categories were measured using a 4-point Likert scale ranging from one (strongly disagree) to four (strongly agree).

#### Adverse life experiences

This study used the Childhood Trauma Questionnaire to measure adverse life experiences or risk in this population. This instrument is a self-report measure that assesses emotional abuse, emotional neglect, and physical neglect. Responses were recorded on a 5-point Likert-type scale, with 1 = "never true" and 5 = "very often true”. This scale demonstrates a Cronbach's alpha of 0.95 [55].

#### Coping

The KidCope was used to assess coping strategies in response to concentrated stress. The KidCope is a measure used in many different global contexts and includes 15-questions to measure ten cognitive and behavioral coping strategies in children and adolescents (Spirito et al., 1988). The original checklist of 15 questions was adapted for this study to include an additional 16th question, “I prayed to feel better” based on qualitative research conducted in Tanzania and the Democratic Republic of Congo [56]. The four-factor structure of the KidCope was validated for these data in a previous study and includes, distraction, resignation, problem-focused and social support coping strategies [57]. Administration of the KidCope first asks adolescents to think of something stressful they have experienced or ongoing stressors and to rate how often they utilized each of the 16 items on a Likert scale (“not at all” = 0 to “all the time” = 1). The KidCope was validated in a study with Tanzanian adolescent orphans and has a Chronbach’s alpha of 0.71 [57].

#### Resilience

The Child and Youth Resilience Measure (CYRM) is a 28-item self-report measure of resilience among young people that has been widely used in a diversity of contexts and translated to more than 20 languages [32]. The 28 item CYRM included 11 items representing a *contextual* resilience subscale. Understandably, this subscale has shown inconsistencies in factor structure in different global contexts [58, 59]. Findings have attributed variation in the CYRM factor structure because of differences in individualistic vs. collectivistic cultures [60, 61]. The Child and Youth Resilience Measure Revised (CYRM-R) is a 17-item, 2-dimensional scale of intra/interpersonal resilience and caregiver resilience subscales that have been validated for use in diverse cultures and contexts [62]. The Chronbach’s alpha for the CYRM in this analytical sample was 0.83.

#### Demographic characteristics

Social and demographic characteristics of participants were collected including age, sex, report of general health, pubertal development status and orphan status.

### Data collection

This study analyzes baseline data from Discover Learning collected in August and October 2020 from non-orphans in the peri urban Temeke District and data collected from orphans in three municipals districts in Dar es Salaam, Tanzania in April and May 2020. The Temeke district in Dar es Salaam, Tanzania is the largest of Dar es Salaam’s three districts and includes both metropolitan urban and rural areas. The study protocol for Discover Learning and these baseline (pre-intervention) data have been described in detail elsewhere, including recruitment, eligibility criteria, data collection procedures and human research protections [63]. In 2020, data were collected from adolescent orphans ages 10 to 15. Participants for this study were recruited in collaboration with our local Tanzania partner, Health for a Prosperous Nation (H-PON) in Tanzania and youth-serving orphanages. The city of Dar es Salaam has five districts: Kinondoni, Ubungo, Ilala, Temeke and Kigamboni. H-PON identified youth orphanages in three peri-urban districts: Ubungo, Ilala and Temeke. Within the city, Ubungo is in the northwest, Ilala in the center, and Temeke in the southeast. Orphanages were introduced to the study and objectives, including consent/assent procedures. Orphanages that agreed to participate were included in the study and research staff obtained permission from the Ministry of Health and Social Welfare responsible for the orphanage to obtain approval to complete research activities. All adolescents ages 10 to 15 agreed to participate and provided assent were included in the study. Data was initially collected to support several objectives including an understanding and measurement of Utu. In this article, we examine the culturally grounded construct of Utu and associated relationships with mental health and well-being measures within these two samples. Comparing orphan and non-orphan groups broadens the understanding of Utu within different populations.

### Data analysis

First, descriptive statistics were calculated for each item measure included in the Utu scale for orphans and non-orphans. Differences between the samples were compared. Second, confirmatory factor analysis (CFA) was executed to test our hypothesis that a five-factor model would best fit these data on the orphan and non-orphan samples, and the full analytic sample. The following conventional criteria used to evaluate goodness of fit of the CFA [64] included chi-square test (model vs. a baseline p ≤ 0.05); the Comparative Fit Index (CFI) values ≥ 0.95 [65]; the Tucker Lewis Index (TLI) values ≥ 0.95 [66]; the Standardized Root Mean Square Residual (SRMR) values ≤ 0.08 [67]; the Root Mean Square Error of Approximation (RMSEA) values ≤ 0.06 [68]; Akaike’s Information Criterion (AIC) [69]; and Bayesian Information Criterion (BIC) [70]. To estimate the internal consistency of scales, researchers calculated Cronbach’s alpha, McDonald’s omega coefficient and item-test correlations for the entire analytical sample.

## Results

### Demographic characteristics of the sample

A total of 333 non-orphan adolescents and 186 orphans completed the survey and were included in the analytical sample. Table 1 reports key demographic characteristics of the analytic sample. 256 males (49.2%) and 263 females (50.6%) with a mean age of 11.5 (SD = 0.7) in the non-orphan sample and 14.5 (SD = 1.9) in the orphan sample. The average household size for the study sample was 6.3 (SD = 2.6).

**Table 1:** Demographic characteristics of analytic sample

	Non-orphans		Orphans		Total	
	n	%	n	%	n	%
Total number of participants surveyed	333	100	186	100	519	100
Sex						
Male	151	45.3	105	56.5	256	49.2
Female	182	54.7	81	43.6	263	50.6
Age						
10	21	6.3	2	1.1	24	4.6
11	133	39.9	6	3.2	139	26.8
12	169	50.8	17	9.4	186	35.8
13	10	3.0	22	11.8	32	6.2
14	–	–	28	15.1	28	5.4
15	–	–	18	9.7	18	3.4
16	–	–	25	13.4	25	4.8
17	–	–	27	14.5	27	5.2
Mean Age (SD)	11.5	0.7	14.5	1.9	12.4	1.8
Health						
General Health Scale (1–4)	2.3	0.5	2.3	0.6	2.3	0.5
Pubertal Development Scale Girls (0–3)^2^	1.5	1.1	1.8	1.1	1.6	1.1
Pubertal Development Scale Boys (0–3)^2^	1.1	1.1	1.3	1.0	1.1	1.1

Mean scores for each item included in the Utu measure are listed by dimensional construct, reported separately for non-orphan and orphan samples and the total sample. Differences between the orphan and non-orphan sample were examined at the item level and are listed in Table 2. Chronbach’s alpha was used to examine internal consistency of each subscale and demonstrated adequate to excellent reliability; *Resource Sharing* (α = 0.73), *Respect and Dignity* (α = 0.82), *Group solidarity* (α = 0.87), *Collectivism* (α = 0.65), *Compassion* (α = 0.86), and for the total Utu measure (α = 0.94). McDonald’s Omega was calculated (0.94) and confirmed reliability of the Utu scale.

**Table 2:** Utu measurement scale item means by orphan status

Dimension and Items	Non-orphans (n = 333)	Orphans (n = 186)	Total (n = 519)	p-value
*Resource Sharing* (α = 0.73)				
1. I share the little that I have with friends and family	2.31 (0.61)	2.43 (0.54)	2.35 (0.58)	0.029*
2. I sacrifice my time for the good of friends and family	2.38 (0.57)	2.39 (0.54)	2.38 (0.56)	0.837
3. Sharing my difficulties with others makes me feel strong	2.30 (0.61)	2.31 (0.68)	2.31 (0.63)	0.976
*Respect and Dignity* (α = 0.82)				
4. I greet my parents and teachers whenever I see them	2.43 (0.52)	2.51 (0.51)	2.46 (0.52)	0.104
5. My parents and teachers expect me to respect their decisions	2.40 (0.57)	2.48 (0.50)	2.43 (0.55)	0.165
6. My parents and teachers treat me with respect and dignity	2.35 (0.59)	2.38 (0.54)	2.36 (0.57)	0.524
*Group solidarity* (α = 0.87)				
7. I have the support of others when I need it	2.39 (0.51)	2.41 (0.51)	2.40 (0.51)	0.878
8. I do helpful things that will benefit me and others I know	2.37 (0.51)	2.45 (0.51)	2.40 (0.51)	0.079
9. When something unfortunate happens to me, others help me out	2.38 (0.53)	2.42 (0.59)	2.40 (0.55)	0.452
*Collectivism* (α = 0.65)				
10. It is my duty to take care of my family and friends even if I have to sacrifice what I want	2.29 (0.58)	2.30 (0.65)	2.30 (0.60)	0.989
11. Being a valuable team player is more important to me than my personal identity	2.14 (0.66)	2.11 (0.78)	2.13 (0.71)	0.658
12. The wellbeing of my friends and loved one is important to me	2.32 (0.50)	2.51 (0.50)	2.38 (0.51)	< 0.001***
*Compassion* (α = 0.86)				
13. I think about and come up with helpful ways for my friends and family to cope with distress	2.25 (0.61)	2.40 (0.56)	2.31 (0.59)	0.012*
14. I direct attention to what is likely to be helpful to others	2.33 (0.53)	2.44 (0.50)	2.38 (0.52)	0.025*
15. I take actions and do things that will be helpful to others	2.35 (0.53)	2.40 (0.53)	2.37 (0.53)	0.366
16. I express feelings of support, helpfulness, and encouragement to others	2.35 (0.03)	2.44 (0.50)	2.38 (0.51)	0.047*
Total Utu Score (α = 0.94)	37.41 (6.26)	38.38 (6.55)	37.74 (6.37)	0.094

p-values: * < 0.05; ** < 0.01; *** < 0.001

### Confirmatory factor analysis on Utu scale measure

We completed confirmatory factor analysis on the analytic sample to assess the hypothesized 5-factor dimensional structure of the Utu measure for Non-Orphans, Orphans and the Total Sample. Results of the CFA for both non-orphan and orphan samples indicated adequate to excellent fit indices and the full analytic sample indicated excellent model fit; CFI = 0.98; TLI = 0.97; RMSEA = 0.046; SRMR = 0.024; chi2 = 202.7, p < 0.001; AIC = 9593.8; BIC = 9843.2 (Table 3).

**Table 3:** Summary of CFA fit indices

Sample	X^2^	Df	p-value	CFI	TLI	AIC	BIC	SRMR	RMSEA
Non-Orphan	161.5	94	0.000	0.98	0.97	6168.1	6389.0	0.031	0.046
Orphan	229.6	94	0.000	0.94	0.92	2967.0	3153.8	0.038	0.088
Total Sample	202.7	94	0.000	0.98	0.97	9593.8	9843.2	0.024	0.046

CFI: Comparative Fit Index; TLI: Tucker Lewis Index; SRMR: Standardized Root Mean Square Residual; RMSEA: Root Mean Square Error of Approximation; AIC: Akaike’s Information Criteria

Mean Utu dimensions by orphan status and gender were analyzed (Table 4). No statistically significant differences were found among boys in the orphan and non-orphan sample. No significant differences between girls and boys were found in the non-orphan or orphan sample.

**Table 4:** Mean Utu dimension response by orphan status and gender

Utu dimensions	Non-orphans			Orphans		
	Boys	Girls	p	Boys	Girls	p
	Mean (SD)	Mean (SD)		Mean (SD)	Mean (SD)	
Resource Sharing	10.1 (1.5)	10.0 (1.3)	0.521	7.0 (1.3)	7.3 (1.6)	0.298
Respect and Dignity	10.3 (1.5)	10.1 (1.4)	0.298	7.2 (1.3)	7.6 (1.4)	0.107
Group Solidarity	10.3 (1.4)	10.1 (1.3)	0.179	7.2 (1.4)	7.5 (1.4)	0.152
Collectivism	9.9 (1.5)	9.7 (1.2)	0.116	6.9 (1.4)	7.0 (1.6)	0.614
Compassion	22.0 (1.5)	21.9 (1.3)	0.304	22.2 (1.4)	22.4 (1.5)	0.480
Total Utu Score	37.9 (6.6)	37.0 (5.9)	0.144	37.9 (6.3)	39.1 (6.9)	0.206

p-values: * < 0.05; ** < 0.01; *** < 0.001

All key variables were compared by orphan status including adverse life experiences, coping strategies, Utu, and the primary outcome measure of Resilience (Table 5). For adverse life experiences, we examined total adverse life experiences and the subdomains emotional abuse, emotional neglect and physical hardship. Orphans had lower mean scores of emotional neglect compared to non-orphans (t = 4.95; p ≤ 0.001), physical hardship (t = 4.69; p ≤ 0.001), and total adverse life experiences (t = 3.58; p ≤ 0.001). Differences in dimensions of coping strategies were found between orphans and non-orphans. Orphans used more distraction coping (t = -4.93; p ≤ 0.001) and more social support coping (t = 6.38; p ≤ 0.001) but less problem focused coping (t = 2.18; p = 0.030) in comparison to non-orphans. No statistically significant differences were found between orphans and non-orphans for Utu total mean score or any of the five subdomains except for the compassion domain where orphans had higher mean compassion scores than non-orphans (t = -2.62; p = 0.009).

**Table 5:** Key Variables by Orphan Status

	Non-orphan		Orphan	
	Mean (SD)	Mean (SD)	t	p-value
*Adverse Life Experiences*				
Emotional Abuse	0.97 (0.89)	1.10 (0.80)	−1.76	0.079
Emotional Neglect	0.76 (0.81)	0.43 (0.61)	4.95	< 0.001***
Physical Hardship	0.75 (1.11)	0.34 (0.61)	4.69	< 0.001***
ALE Total	2.48 (2.05)	1.87 (1.50)	3.58	< 0.001***
*Intra/Interpersonal Assets*				
Distraction Coping	2.20 (0.89)	2.61 (0.94)	− 4.93	< 0.001***
Resignation Coping	2.12 (0.81)	2.13 (0.65)	− 0.18	0.859
Problem Focused Coping	2.52 (0.89)	2.34 (1.02)	2.18	0.030*
Social support Coping	2.77 (0.83)	3.23 (0.74)	-6.38	< 0.001***
*Collective Asset*				
Resource Sharing	7.00 (0.74)	7.13 (0.11)	− 0.99	0.325
Respect and Dignity	7.20 (0.75)	7.37 (0.10)	− 1.41	0.160
Group Solidarity	7.16 (0.07)	7.28 (0.11)	− 0.99	0.324
Collectivism	6.76 (0.07)	6.92 (0.11)	− 1.24	0.215
Compassion	21.94 (0.07)	22.28 (0.11)	− 2.62	0.009**
Utu Total	37.41 (6.25)	38.4 (6.5)	− 1.68	0.094
*Primary Outcome*				
Resilience	78.45 (8.29)	79.8 (6.89)	−1.93	0.055

*p < 0.05; **p < 0.01; ***p < 0.001

Prior to fitting a structural equation model, regression analysis was used to assess key variables and sub-dimensions on resilience subscales; intra/interpersonal resilience, and caregiver resilience (Table 6). Results of robust regression analyses on resilience dimension indicated that orphans held higher intra/interpersonal resilience in comparison to non-orphans (β = 1.82; p = 0.012). Adolescents who had experienced emotional neglect had lower intra/interpersonal resilience (β = −081; p = 0.023) and caregiver resilience (β = − 0.66; p = 0.010). Adolescents who scored higher in the Resource Sharing subdimension of Utu had higher intra/interpersonal resilience after adjusting for covariates (β = 086; p = 0.004).

**Table 6:** Multivariable robust regression on resilience dimensions by independent variables

	Intra/Interpersonal resilience			Caregiver resilience		
	Coef.	Robust SE	p-value	Coef.	Robust SE	p-value
Demographic Characteristics						
Age	−0.30	0.44	0.502	−0.08	0.31	0.790
Sex	−0.11	0.17	0.509	−0.22	0.14	0.116
Orphan Status^1^	1.82	0.72	0.012*	−0.06	0.57	0.911
Adverse Life Experiences						
Emotional Abuse	−0.64	0.32	0.047	−0.25	0.25	0.306
Emotional Neglect	−0.81	0.36	0.023*	−0.66	0.26	0.010*
Physical Neglect	−0.17	0.27	0.531	−0.28	0.21	0.193
Coping Strategy						
Distraction	0.13	0.28	0.634	0.13	0.22	0.570
Resignation	0.20	0.32	0.531	−0.18	0.25	0.457
Problem Focused	0.00	0.26	0.986	0.16	0.20	0.421
Social Support	0.15	0.35	0.656	0.13	0.29	0.659
Utu						
Resource Sharing	0.86	0.29	0.004**	0.42	0.22	0.053
Respect/Dignity	−0.36	0.30	0.234	−0.13	0.18	0.474
Solidarity	−0.34	0.35	0.332	0.07	0.22	0.752
Collectivity	0.30	0.15	0.051	−0.21	0.15	0.169
Compassion	0.18	0.31	0.551	0.21	0.23	0.375

*p < 0.05; **p < 0.01; ***p < 0.001

**Fig. 1:**
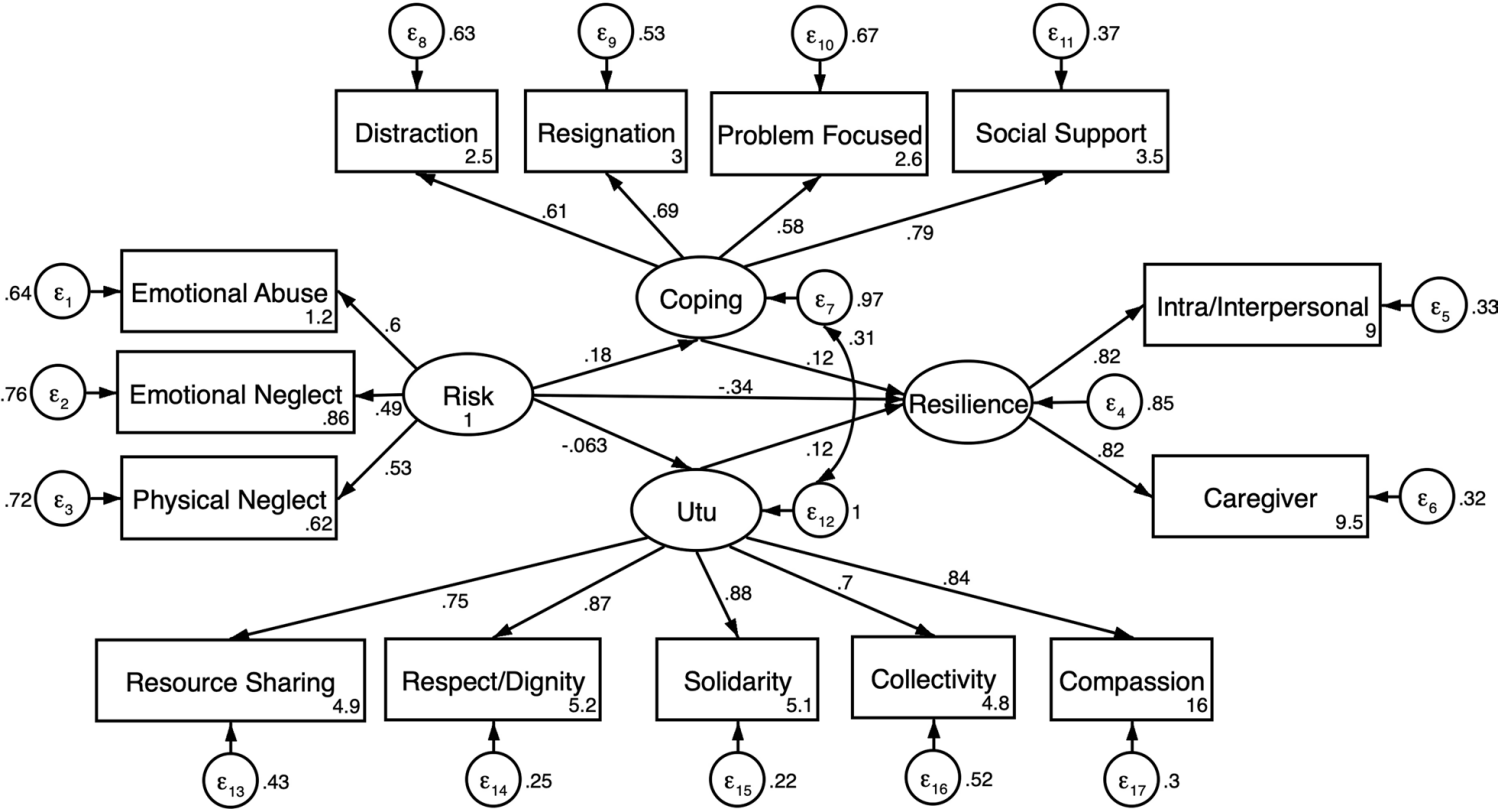
Structural equation model of risk, internal adaptive assets, community adaptive assets and resilience. Note. CFI = 0.97; TLI = 0.96; SRMR = 0.044; RMSEA = 0.048

The latent variables risk, coping, Utu, and resilience were fitted in a structural equation model to examine measurement models for each latent variable and structural paths between latent variables (Fig. 1). The structural equation model fit indices indicated excellent fit (CFI = 0.97, TLI = 0.96, RMSEA = 0.048, SRMR = 0.043) for these data. Standardized path coefficients between latent variables with robust standard errors and p-values are listed in Table 7. Adverse life experiences were associated with lower resilience (β = − 0.35; p < 0.001). Both use of coping strategies (β = 0.13; p = 0.038) and Utu (β = 0.12; p = 0.026) were associated with higher reported resilience. Adverse life experiences were significantly associated with higher use of coping strategies (β = 0.19; p = 0.004) but was not associated with Utu (β = − 0.06; p = 0.307). Covariance parameter estimates between Utu and coping strategies were significant (β = 0.31; p ≤ 0.001). The measurement model results indicate all factor dimensions for each latent variable were significant (p ≤ 0.001).

**Table 7:** Standardized path coefficients associated with resilience among adolescent orphans

Structural Model	Standardized coefficient	Robust SE	z	p >|z|	95% Confidence Interval	
*Resilience*						
Adverse life experiences	− 0.35	0.06	−5.48	< 0.001***	− 0.47	0.22
Coping	0.13	0.06	2.07	0.038*	0.01	0.25
Utu	0.12	0.05	2.23	0.026*	0.01	0.22
*Coping Strategies*						
Adverse life experiences	0.19	0.08	2.86	0.004**	0.06	0.32
*Utu*						
Adverse life experiences	− 0.06	0.06	−1.02	0.307	− 0.18	0.06
*Covariance*						
e. Coping, e. Utu	0.31	0.05	6.12	< 0.001***	0.21	0.40
*Measurement Model*						
*Adverse Life experiences*						
Emotional Abuse	0.60	0.6	10.8	< 0.001***	0.49	0.71
Emotional Neglect	0.49	0.53	9.26	< 0.001***	0.39	0.60
Physical Neglect	0.53	0.53	9.99	< 0.001***	0.39	0.60
*Coping Strategies*						
Distraction	0.66	0.03	19.42	< 0.001***	0.59	0.73
Resignation	0.62	0.04	17.20	< 0.001***	0.55	0.69
Problem Focused	0.62	0.04	17.42	< 0.001***	0.55	0.69
Social Support	0.71	0.03	21.51	< 0.001***	0.65	0.78
*Utu*						
Resource Sharing	0.75	0.02	35.83	< 0.001***	0.72	0.80
Respect/Dignity	0.87	0.01	63.19	< 0.001***	0.84	0.89
Solidarity	0.88	0.01	69.35	< 0.001***	0.84	0.89
Collectivity	0.70	0.03	28.43	< 0.001***	0.65	0.74
Compassion	0.84	0.02	32.64	< 0.001***	0.81	0.87
Resilience						
Intra/Interpersonal	0.82	0.06	14.14	< 0.001***	0.71	0.94
Caregiver	0.82	0.06	14.13	< 0.001***	0.71	0.94

*p < 0.05 **p < 0.01 ***p < 0.001

X^2^ = 159.579; p = 0.000; CFI = 0.97; TLI = 0.96 RMSEA = 0.048 SRMR = 0.043; AIC = 22,588; BIC = 22,794

**Fig. 2:**
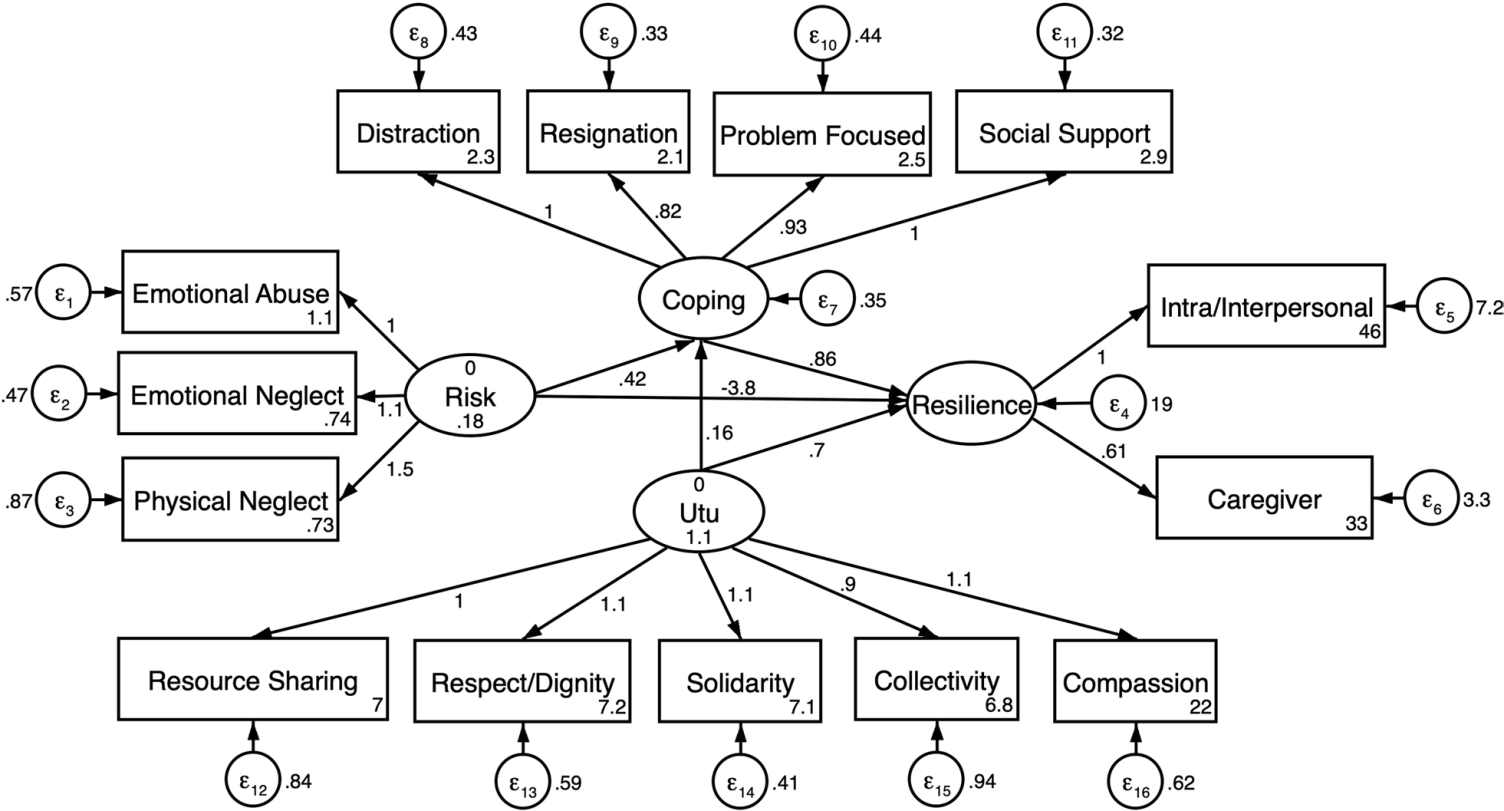
Revised structural equation model of risk, internal adaptive assets, community adaptative assets and resilience. Note. CFI = 0.97; TLI = 0.96; SRMR = 0.044; RMSEA = 0.048

After observing that the structural path from risk to Utu was not significant, but the correlation between Utu and coping latent variables was significant, we examined modification indices that suggested a significant path from Utu to coping. Therefore, a second structural equation model was fit removing the path from risk to Utu latent factors and adding a path from Utu to coping (Fig. 2). Evaluation of goodness of fit indices indicate excellent model fit (X^2^ = 160.62; p < 0.001; CFI = 097; TLI = 0.96; SRMR = 0.44; RMSEA = 0.048).

Structural path coefficients of the refitted structural equation model are presented in Table 8. In the revised SEM all structural path coefficients were statistically significant (in contrast to the initial SEM), and the added path from Utu to coping was positively associated with use of coping strategies (β = 0.17; p ≤ 0.001). All paths from subscale dimension measures to latent variables were significant at the p ≤ 0.001 level.

**Table 8:** Standardized path coefficients associated with resilience among adolescent orphans

Structural Model	Standardized coefficient	Robust SE	z	p >|z|	95% Confidence Interval	
*Resilience*						
Adverse life experiences	−0.35	0.06	−5.47	< 0.001***	−0.47	−0.22
Coping	0.13	0.06	2.08	0.038*	0.01	0.25
Utu	0.13	0.05	2.46	0.014*	0.03	0.23
*Coping Strategies*						
Adverse life experiences	0.21	0.06	3.28	0.001**	0.08	0.33
Utu	0.29	0.05	6.04	< 0.001***	0.20	0.39

*p < 0.05; **p < 0.01; ***p < 0.001

X^2^ = 160.62; p = 0.000; CFI = 0.97; TLI = 0.96 RMSEA = 0.048 SRMR = 0.044; AIC = 22,587; BIC = 22,789

## Discussion

This study is the first of our knowledge to develop a scale measure of Utu in Tanzania for adolescents. While Utu is a culturally defined and grounded concept, similar constructs exist across sub-Saharan Africa. Utu, and the values it represents are a unique community asset that has the potential to improve individual coping strategies and intra/interpersonal and collective resilience.

Exploration of differences by gender and orphan status were evaluated prior to fitting regression and structural equation models. There were no significant differences in Utu subdimensions by gender. Testing by orphan status was also insignificant for all subdimensions except for compassion, where orphans demonstrated significantly higher subscale scores in comparison to non-orphans. It is hypothesized that orphans may demonstrate higher compassion scores than nonorphans because of their living context where orphans live and form friendships with a diverse pool of peers. This finding is also consistent with previous research that found higher levels of emotional empathy among orphans in comparison to non-orphans [71]. The lack of differences in other dimensions of Utu by gender and orphan study resonate with the philosophical underpinnings of Utu that holds humanness as essential and protected irrespective gender, race, tribal, religious or political affiliations [72]. Interventions that seek to strengthen Utu in communities may be positioned well to integrate equity enhancing objectives in ways that would be culturally acceptable and endorsed in communities. For example, research from Zanzibar on a women’s savings cooperate of migrants from the Tanzanian mainland, found that *Umoja*, a concept that roughly translates to “unity” and is closely tied to Utu, has been increasingly used to negotiate gender justice[73]. Umoja, “maintains relational dignity among members and structurally mitigates within-group inequities” by prioritizing collectivity and allowing women to negotiate rights based gender justice without directly confronting patriarchal social structures [73]. An approach that leverages Utu as a positive cultural asset can be equity enhancing and a culturally acceptable approach, that defines equity as intersectional and relational, and outside of a colonial paradigm and western conceptualizations of gender rights [73]. Similarly, interventions that explicitly integrate Utu learning and practice may be an effective approach to promote gender equity by changing belief and behaviors of adolescents [74].

In the fitted structural equation model, adverse life experiences were associated in a direct path to resilience but were also significantly associated with increased use of coping strategies which mediated the effect of stress on resilience. Use of coping strategies and their associated adaptive capacity are situational and context dependent [75, 76]. Results from previous studies support the existing theory that experiences of stress motivate individuals to use coping strategies to adapt to stressors [77, 78]. Additionally, the positive effect of coping strategy use varies widely in different contexts [79]. Theory posits that learning to use coping strategies effectively changes over time as the adolescent develops [80]. The choice of coping strategy deployed, and frequency of their use are associated with stress exposure, type and duration [81]. In this study adolescents who reported higher use of coping strategies demonstrated higher resilience. Orphans also had higher intra/interpersonal resilience capacities than non-orphans. These results indicate that orphans may have higher adaptive capacities related to being cared for in a large, social context where peers become ‘family’ in the absence of a traditional family structure.

Higher levels of Utu were significantly associated with higher resilience in these data. However, unlike use of coping strategies, the structural path between adverse life experiences and Utu were not significant. This suggests that as a collective asset, Utu is not directly associated or contingent on levels of risk posed by adverse life experiences. In the revised model that removed the non-significant path from risk to Utu and added a path from Utu to coping, goodness of fit indices indicated excellent model fit. The second model indicates that Utu is positively associated with resilience, independent of risk exposure, and with increases in use of coping strategies. The positive relationship between Utu and coping strategies can be explained by the fact that Utu generates reciprocal, reinforcing support networks. The greater number of peer and community supports available to adolescents may increase their use of particular coping strategies such as using social supports to help solve problems or engaging with peers to distract from stressors. The finding that Utu is significantly related to both intra/interpersonal and collective resilience underscores the importance of Utu as a promotive and protective factor. Research in other sectors such as business, where utu-ubuntu business models explain the benefits of utu to economic livelihoods because Utu is defined by expressions of interconnection and reciprocity that create self-regulating networks prioritized over individualistic growth [82]. For these reasons, interventions that target cultivation and practice of Utu may be particularly effective in building adolescent and community resilience to concentrated adversity such as civil disorder, natural disasters or global pandemics [72, 83].

Programs that seek to integrate promotion of Utu should consider implementation of multi-level interventions (individual, peer, family and community levels). A previous evaluation of the Discover Learning intervention in Tanzania with early adolescents ages 10–11 indicated that adolescents who received individual, peer, family and community-engaged components had greater effect size changes on positive mental health and wellbeing outcomes in comparison to study arms that did not include these components [74]. This study incorporated culturally significant traditions and artifacts by asking adolescents to engage with the community to design, produce and present a *Kanga,* a printed cotton fabric that often includes a culturally meaningful proverb, and is traditionally gifted or passed down through generations to families or members of the community as a symbol of collective unity and wisdom [74]. Other studies reflect the value of leveraging collective representations or symbols of community, that help build social cohesion, trust and encourage helping behaviors in response to stressors [17]. Other promising approaches to enhance collective assets include community-based participatory approaches that seek to promote trust, mutual understanding and collective problem solving [84–86]. A community-based participatory approach to designing Utu intervention components can leverage community and collective social experiences to translate research to practice effectively and enrich programs [85]. Research indicates that including community components can improve mental health care in low and middle income countries, decrease violence and increase community resilience [87, 88]. In a study in Tanzania that measured sociocultural variables meant to capture components of Utu, it was found that several of these variables were related to risk of homicide in Dar es Salaam [89].

A growing body of research recognizes that the concept of Utu is important to designing culturally relevant and efficacious interventions in Tanzania and in other sub-Saharan countries with similar concepts [15, 89–91]. As a collective asset, Utu can be targeted as a modifiable protective factor during adolescence to promote resilience. While research examining Utu is emerging in several fields, a measure designed to capture the unique value system of Utu can be incorporated into analytical models to capture the protective and promotive attributes of this cultural asset.

This study has some limitations. It includes follow-up data from adolescents who participated in Discover Learning, a social emotional learning intervention delivered from July to August 2019. Articles detailing the study protocol of the 2019 Discover Learning intervention and results are available in peer review publications [63, 74, 92]. While the social emotional learning intervention did not include Utu content, it is possible that improvements in social emotional skills and mindsets resulting from participation in Discover biased results for the non-orphan sample. There were differences in the mean age between the orphan and non-orphan sample because the non-orphan sample was drawn from a study targeting early adolescents [63].

## Conclusions

This study resulted in a novel measure of Utu, a community asset that is associated with higher capacities for resilience and lower psychopathology in both orphan and nonorphan populations. The concept of Utu while culturally grounded, transcends country borders and has relevance to several sub-Saharan countries. Implications for adolescent programs that aim to improve resilience may benefit from integrating Utu into programmatic content. Globally, multi-level interventions should consider measurement and evaluation of culturally defined collective assets that can be leveraged to mitigate risk and promote wellbeing.

## Data Availability

The datasets generated and/or analyzed during the current study are not publicly available due to the sensitive age of the study participants (10–15-year-old). These data are available from the corresponding author on reasonable request. The author will vet requests to be certain that appropriate IRB approvals and data safety guidelines are in place before distribution.
